# Sustainable Process for Tortilla Production Using Ohmic Heating with Minimal Impact on the Nutritional Value, Protein, and Calcium Performance

**DOI:** 10.3390/foods12183327

**Published:** 2023-09-05

**Authors:** Aurea K. Ramírez-Jiménez, Rubén Cota-López, Eduardo Morales-Sánchez, Marcela Gaytán-Martínez, Héctor Eduardo Martinez-Flores, María de la Luz Reyes-Vega, Juan de Dios Figueroa-Cárdenas

**Affiliations:** 1 Tecnologico de Monterrey, School of Engineering and Science, Avenida Eugenio Garza Sada 2501 Sur, Monterrey NL 64849, Nuevo León, Mexico; aramirezj@tec.mx; 2 Instituto Politécnico Nacional, CICATA-IPN Unidad Querétaro, Cerro Blanco No. 141, Col. Colinas del Cimatario, Santiago de Querétaro CP 76090, Querétaro, Mexicoemoraless@ipn.mx (E.M.-S.); 3 Posgrado en Ciencia y Tecnología de los Alimentos, Research and Graduate Studies in Food Science, School of Chemistry, Universidad Autónoma de Querétaro, Cerro de las Campanas S/N, Col. Centro, Santiago de Querétaro CP 76010, Querétaro, Mexico; 4 Facultad de Químico-Farmacobiología, Universidad Michoacana de San Nicolás de Hidalgo, Tzintzuntzan 173, Col. Matamoros, Morelia CP 58240, Michoacan, Mexico; 5 Department of Research and Graduate Studies, Universidad Autónoma de Querétaro, Cerro de las Campanas S/N, Col. Centro, Santiago de Querétaro CP 76010, Querétaro, Mexico; 6 CINVESTAV del IPN, Unidad Querétaro, Libramiento Norponiente No. 2000, Real de Juriquilla, Santiago de Querétaro CP 76230, Querétaro, Mexico

**Keywords:** protein efficiency ratio, calcium uptake, bone crystallinity, nixtamalization process, ohmic heating, tortilla processing, nutritional value

## Abstract

The nixtamalization process used for tortilla production entails extended processing time and generates pollutant effluents. Ohmic heating (OH) is an emerging technology that uses an alternating electric current for rapid and uniform food heating and mitigates effluent concerns. However, gaps exist in nutrient bioavailability studies. In this work, we assessed OH’s impact on tortilla nutritional value, protein, and calcium using a rat model. Twenty-five male Wistar rats were fed one of four diets for 21 days: raw corn (RC) as an experimental control, OH-processed tortillas (OHTs), traditionally processed tortillas (TPTs), commercial tortillas (CTs), and a casein diet (CD) as a growth control. Despite similar protein and macronutrient profiles, OH significantly enhanced insoluble fiber content. The weight gain sequence was OHTs > TPTs > CTs > RC. OHTs exhibited superior protein digestibility (88.52%), which was 3% higher than other diets. The serum albumin (2.63–2.73 g/dL) indicated moderate malnutrition due to the tortilla’s lower protein content. Nonetheless, the protein efficiency ratio (1.2–1.74) showed no significant difference from TPTs. Bone characteristics and fracture strength resembled the tortilla-fed groups, surpassing RC. X-ray diffraction and scanning electron microscopy confirmed that the OHT and TPT diets improved male rat bone thickness and crystallinity. The findings suggest the potential for OH as an eco-friendly tortilla production method, maintaining nutritional value comparable to traditional methods.

## 1. Introduction

Tortillas are a food product that is consumed in many Latin American countries and has an increasing market in the USA [1]. These products are considered as a primary source of nutrients such as carbohydrates, proteins, and calcium, especially in rural areas [2]. The traditional nixtamalization process used to create tortillas consists of alkaline cooking maize grains with lime (Ca[OH]_2_), followed by a 12 h steeping step and thorough washing to remove the pericarp. This process improves the protein quality by selectively enhancing the amino acid profile. During thermo-alkaline cooking, zein (a nutritionally poor protein due to its deficiency in lysine and tryptophan) decreases in solubility, whereas glutelins (proteins with a higher nutritional value) increase in solubility [3,4]. This effect modifies the protein efficiency and performance and the bioavailability of essential amino acids. In this sense, Maya-Cortés et al. [5] reported a 25% increase in the protein efficiency ratio (PER) and a 17% increase in weight gain of weaning rats fed tortillas compared with rats fed raw corn (RC).

Moreover, several studies have reported that the calcium content increased by up to 400% of that in corn grains [6,7], as well as its bioavailability, due to the Ca[OH]_2_ added during the cooking process. Nixtamalized products represent a significant source of calcium for many Latin American populations [8], as demonstrated in a study conducted with Mexican women who consumed nixtamalized tortillas for 12 days [9]. Calcium absorption showed a 25-fold increase compared with women who consumed non-nixtamalized tortillas.

The traditional nixtamalization process has some disadvantages, such as long soaking and cooking times, excessive water wastage, and the generation of polluting effluents, where large amounts of nutrients are lost [10]. The cooking water used for maize nixtamalization is known as “nejayote.” This alkaline liquid, when improperly discarded, exerts a significant environmental impact due to its high chemical and biochemical oxygen demand. Approximately 14.4 million m^3^/month are estimated to be discharged into drainage systems [11]. Furthermore, it has been reported that certain nutrients and bioactive molecules such as proteins, fiber, and antioxidants leach into the nejayote, thereby reducing the nutritional quality of tortillas [10]. Moreover, the industrialization of nixtamalization for tortilla creation compromises the nutritional quality of tortillas, generating products with lysine, tryptophan [12], and calcium [9,13] deficiencies. Several studies have been conducted using alternative and ecological methods to create tortillas; however, the physicochemical, textural, and sensory properties are often affected. For example, tortillas obtained using extrusion have high starch gelatinization, which consumers perceive as excessively sweet, which is a non-desirable taste in tortillas [14].

Ohmic heating (OH) is an emerging technology that allows for the rapid and uniform heating of foods [15,16,17]. Using this method, an alternating electrical current (<1000 V/cm) is applied to the food matrix [18]. Consequently, the material is internally heated from the core to the outer material’s surface due to the food’s electrical resistance [19,20]. This feature is the main innovation of OH, making it an energy-efficient process suitable for the rapid and uniform production of food products [21]. When this technology is used, food is not over-processed; therefore, nutrient loss is minimized [17,19,22,23]. OH technology is also known as moderate electric fields [18,21], electroheating [24], and capacitive dielectric heating [25].

Previous studies have used OH nixtamalization to obtain corn flour and tortillas [22,26]; however, these studies were focused on the physicochemical characteristics of the products obtained. Ménera-Lopez et al. [26] evaluated a batch OH process using different conditions to obtain corn flour and tortillas. The products obtained using this method showed physicochemical characteristics similar to traditionally produced tortillas. Previously, we reported that corn flour obtained using OH preserves its bioactive compounds, including fiber and phenolic compounds [23], with a cooking time of around 5 min.

Because tortillas are a primary source of protein and calcium for some populations, especially in rural areas [2], the utilization of new technology must produce at least the same protein and nutritional quality as the traditional process. This has substantial importance considering that several countries in Africa and South America exhibit predominantly low calcium intake. In certain regions, up to 70% of the population does not meet the minimum calcium intake, leading to deficiencies in the optimal development of bone mass and bone health in adults. Moreover, this concern extends to malnutrition resulting from protein deficiencies, particularly affecting the pediatric population in these regions [27,28]. In 2020, the prevalence of stunted growth in children under 5 years of age in Latin America and the Caribbean stood at 11.3%, approximately 10 percentage points below the global average [29].

The aim of this study was to determine whether OH could be used for tortilla production without altering the nutritional quality, especially concerning proteins and calcium. This study evaluated the proximate composition and protein profile of tortillas. Moreover, food efficiency, protein efficiency, and digestibility were measured, and the calcium performance in bones, including the femur fracture resistance, physical bone dimensions, and bone X-ray diffraction patterns, were analyzed in an in vivo study using male Wistar rats. The outcomes measured in each analysis were compared with those measured for a raw corn (RC) diet, tortillas produced using traditional nixtamalization (TPT), and commercial tortillas (CTs).

## 2. Materials and Methods

### 2.1. Biological Materials

Corn (*Zea mays*) grown and harvested in Sinaloa, México, was used for flour production. The corn grains were manually cleaned to remove dust and debris and stored in polyethylene containers at 4 °C.

### 2.2. Flour and Tortilla Preparation

#### 2.2.1. Flour Obtained using the Traditional Nixtamalization Process

Corn grains were added to a lime (Calidra, Santiago de Querétaro, Mexico) solution (2 L water/kg corn and 10 g lime/kg corn), cooked in a stainless-steel steamer at 90 °C for 25 min, and allowed to stand for 12 h [22]. The soaked grains (nixtamal) were washed once to remove excess lime and pericarp residues and ground in a stone miller (FUMASA, mod. US-25) to obtain fresh dough (*masa*). The *masa* was dehydrated using a flash-type dryer (Cinvestav-AV, M2000, Santiago de Querétaro, Querétaro, Mexico) with a capacity of 50 kg/h) at a temperature of 270 ± 5 °C and an outlet temperature of 40 °C. Afterward, the sample was ground using a hammer mill (PULVEX 200) fitted with a 0.8 mm aperture mesh (Model 200).

#### 2.2.2. Flour Obtained using Ohmic Heating Nixtamalization

One kilogram of grains was ground using a hammer mill (Pulvex S.A. de C.V.) equipped with a 3 mm mesh. The obtained raw flour was mixed with 3 g calcium hydroxide/kg corn (*w*/*w*). Water was added until 50% moisture was reached using a commercial mixer (Kitchen Aid model K45SS; St. Joseph, MI, USA). The mixture was passed through a continuous ohmic cooker [30], consisting of a screw conveyor coupled to a food-grade polycarbonate cell (5 × 5 × 20 cm) equipped with stainless-steel electrodes and a thermocouple for temperature measurements. The applied voltage inside the cell was 120 VAC, and the temperature was controlled using a temperature controller (Watlow, Model 981 Winona, MN, USA). The mixture was fed into the screw conveyor at 20 rpm and heated to 85 °C. An electric field strength (FES) of 25 V/cm was applied at 60 Hz for 3 min [23]. The *masa* obtained was dried using a flash-type dryer (Cinvestav-AV, M2000, Querétaro, Mexico). Afterward, the material was ground again using a hammer mill (PULVEX 200) fitted with a 0.8 mm mesh (Model 200).

#### 2.2.3. Tortilla Elaboration

The corn flour obtained using ohmic heating (OHT), the flour obtained with the traditional process, and a commercial corn flour were used to create the tortillas. The flours were mixed with enough water to obtain dough (“*masa*”) with an adequate consistency to shape the tortillas [22]. The *masa* disks (1.2 × 12.5 mm) were obtained using a tortilla die cutter and cooked at 270 °C. The tortillas were heated for 17 s to form a thin layer, turned over for a further 50 s to form the thick layer, and turned to the original side for another 17 s to allow the tortillas to inflate. The obtained tortillas were coded as tortillas processed using ohmic heating (OHTs), tortillas obtained with the traditional process (TPTs), and commercial tortillas (CTs). Additionally, the commercial flour used to make the commercial tortillas, and raw corn (RC), without any further processing, were used as the controls to compare the nutritional value, protein, and calcium performance of the tortillas. This was completed to confirm whether the OH process achieved an effect on nutrient digestibility/availability at least equivalent to that of the traditional nixtamalization process.

### 2.3. Nutritional Composition

Raw corn (RC) and tortillas obtained using the different nixtamalization processes (including tortillas made from commercial flour) were analyzed for moisture content, fat, ash, protein, and calcium using the methods 934.01, 942.05, 30.10, 32.1.22, and 968.08 of the Association of Official Analytical Chemists (AOAC) [31], respectively. The total dietary fiber was determined using the method described by Prosky et al. [32]. Lysine and tryptophan were quantified according to the method reported by Tsai et al. [33] and modified by Gallicia et al. [34]. The lysine content was calculated using a standard curve and reported according to protein content.

### 2.4. Sequential Extraction of Protein Fractions

Isolation of globulins, albumins, prolamins, and glutelins was performed according to the method reported by Landry, Delhaye, & Damerval [35]. A prior defatting step was performed in the tortillas by mixing each sample with 3 volumes of hexane (J.T. Baker, Phillipsburg, NJ, USA), shaking for 2 h, and centrifuging (12,000 × *g* for 5 min) to remove the solvent. This step was repeated three times, and the residual solvent was removed with a rotary evaporator. The samples were maintained in microcentrifuge tubes at −20 °C until further analyses.

The methodology proposed by Landry et al. [35] was used for the sequential extraction. Globulins and salt-soluble albumins (F1) were extracted twice with 0.5 M NaCl (J.T. Baker, Phillipsburg, NJ, USA) at 4 °C for 30 min with constant agitation. Albumins (F2) were extracted twice with water at 4 °C for 15 min. α-, β-, and δ-zeins (F3) were extracted three times in 55% 2-propanol (J.T. Baker, Phillipsburg, NJ, USA) + 0.6% β-ME (*v*/*v*) (Sigma-Aldrich Co., St. Louis, MO, USA) for 30 min, 30 min, and 15 min, respectively. γ-zeins and glutelin-like proteins (F4) were extracted twice in 0.5 NaCl + 0.6% β-ME (*v*/*v*) in borate buffer pH 10 (0.0125M Na_2_B_4_O_7_ [Sigma-Aldrich Co., St. Louis, MO, USA]) for 15 min. Glutelins (F5) were extracted twice in 0.5% SDS (*p*/*v*) + 0.6% β-ME, pH 10 for 30 min. The non-extractable (insoluble) protein residue (F6) was dissolved in 3 volumes of 10% TCA (Sigma-Aldrich Co., St. Louis, MO, USA) + 2% β-ME/ with continuous agitation in an ice bath for 15 min. All fractions were centrifuged after each extraction step at 12,000 × *g* for 5 min at 4 °C. The protein fractions were concentrated and stored at −70 °C until use. Proteins were quantified with the bicinchoninic acid assay (Pierce BCA protein assay, ThermoFisher Scientific, Waltham, MA, USA) using bovine serum albumin (2 to 2000 μg/mL) as the standard.

### 2.5. SDS-PAGE

Protein fractions were resuspended in 100 μL sample buffer (62.5 mM Tris-HCl [Invitrogen, Burlington, ON, Canada], pH 6.8, 25% glycerol (Sigma-Aldrich Co., St. Louis, MO, USA), 2% SDS, 0.01% bromophenol blue (Sigma-Aldrich Co., St. Louis, MO, USA), 0.4 mL β-mercaptoethanol) to achieve a final concentration of 1 mg/mL. SDS-PAGE was performed on a vertical gel system Mini-Protean^®^ Tetra (Biorad Corporation, Hercules, CA, USA) using 15% polyacrylamide resolving gel and 4% stacking gel. Fifteen microliters of each fraction were loaded into the wells of the resolving gel and run at 80 V for 20 min and 110 V for 1.5 h. The gels were stained with Coomassie R-250 at room temperature (25 °C) for 15 min. A precision plus protein^TM^ dual color standard (Cat. 1610374, Biorad Corporation, Hercules, CA, USA) was used as a molecular ladder. Images were acquired using a ChemiDoc MP Imaging System (Biorad Corporation, Hercules, CA, USA).

### 2.6. Biological Assay

#### 2.6.1. Study Design

Twenty-five male Wistar rats (23–25 days old, body weight 83.68 ± 6.97 g) were used for the experiment. The research protocol was designed and conducted in accordance with the Mexican Official Normativity for the production, care, and use of laboratory animals (NOM-062-ZOO-1999) and the American National Institute of Health. This research was approved by the bioethics committee of the Universidad Michoacana de San Nicolás de Hidalgo (Rec. No. 01-16CE-FAC QFB). The animals were allowed to acclimatize for 4 days in cages before being assigned to the experimental groups. During this period, each animal was fed a commercial diet (5001 Rodent Laboratory Chow 5001, Agroser Purina distributor, Morelia, Michoacán, México). The rats were weighed and randomly assigned to one of the five groups corresponding to the experimental diets (*n* = 5). The animals were housed in individual cages in a room maintained at 22 °C with a 12 h light–dark cycle and fed ad libitum with the experimental diets and water for 21 days. Fecal samples were collected daily during the second and third weeks and stored at −20 °C. After 21 days, the rats were sacrificed using cervical dislocation, and blood samples were obtained using a cardiac puncture; the freshly collected blood was used for serum album analysis. The lower limbs were dissected, the femurs were obtained, and the surrounding tissues were removed. The femurs were used for resistance tests, calcium content, and X-ray diffraction analysis.

#### 2.6.2. Diet Preparation

The experimental design was based on the method described by [5]. Five experimental diets were formulated based on the AIN-93G Diet standard [36]. The composition of the diets is described in Supplementary Table S1. Diets were prepared from RC, CTs, tortillas made using the traditional process (TPTs), and tortillas obtained using OH (OHTs). A control diet containing casein (CD) was prepared as internal control to ensure the experiment was well performed, and to monitor the normal growth of rats. Theoretical values of the proteins were adjusted to 7 g/100 g for the treatment groups; the casein diet (CD) was not adjusted since it was the control. The chemical composition of the experimental diets is shown in Table 1.

#### 2.6.3. Food Intake and Efficiency

Food intake was recorded daily, and the body weight was measured every 7 days starting from day 0. The accumulated food intake and final weight were used for further calculations. Food efficiency was calculated as the total weight gain (after 21 days) divided by the total food intake and expressed as a percentage.

#### 2.6.4. Protein Efficiency Ratio, Apparent Protein Digestibility, and Serum Albumin

The protein efficiency ratio (PER) was measured according to the methodology described by [4]. This is a biological assay that evaluates the quality of a particular dietary protein, measured as the weight gain per gram of protein consumed during the test period. For this study, PER was calculated, as shown in Equation (1). PER values were normalized for all groups using the value assigned to the casein control group as reference, PER = 2.5 (Equation (2)).

(1) $$PER=\frac{Weight\;gain\;(g)}{Food\;intake\;\left(g\right)\;*\;Protein\;(g/100g)}$$

(2) $$Normalization\;PER=\frac{PER\;of\;experimental\;diet}{PER\;of\;CD}$$

Serum albumin was determined using the Bromcresol green colorimetric method (Cat number 1001020, Spinreact S.A., Girona, Spain). For the apparent protein digestibility (APD) analysis, fecal samples were collected during the last 10 days of the study and analyzed for total protein content using method 32.1.22 of the AOAC [31]. The APD was calculated using the following equation.

(3) $$\;APD=\frac{Protein\;consumed\;\left(g\right)-fecal\;protein\;(g)}{Protein\;consumed\;(g)}\times 100$$

#### 2.6.5. Physical Characteristics of Bones

A previously cleaned and dried (45 °C for 24 h) femur from each rat was used to measure the length, thickness, and diameter with a caliper. The weight of each femur was also recorded using an analytical balance.

#### 2.6.6. Resistance Test

A cutting test was performed to determine the femurs resistance in a Texture Analyzer (Brookfield CT3 model, Middleboro, MA, USA) using a knife blade with a 45 °C chisel at a speed of 10 mm/s and 10 cm distance. The fracture strength was determined from the deformation curve and expressed as N.

#### 2.6.7. X-ray Diffraction Pattern

The X-ray diffraction pattern of the femurs was assessed using the method reported by Contreras-Padilla et al. [37] and was performed using a Rigaku Miniflex X-ray diffractometer (DMAX-2100, Rigaku, Tokyo, Japan). The operating conditions were: 35 kV, 15 mA, and a Cu-Kα radiation wavelength of λ = 1.5406 Å. Data were collected from 2 to 30° on the 2θ scale using a step size of 0.02°.

#### 2.6.8. Scanning Electron Microscope (SEM)

A Jeol JSM-6060 LV scanning electron microscope was used in low vacuum mode with the electron beam set to 10 kV. The bones were cut into small pieces (0.5 cm) and immersed and fixed onto acrylic pads. Samples were coated with gold, and the SEM analysis was performed at 33× and 100×.

### 2.7. Statistical Analysis

All chemical determinations were performed in triplicate. The results of the biological tests were obtained from the five rats per group. Differences between the diets were analyzed using a one-way analysis of variance with a level of significance of α = 0.05. The means were compared using the Tukey–Kramer test, and differences were considered significant when *p* < 0.05. Statistical analyses were conducted using JMP^®^, Version *8.0*. SAS Institute Inc., Cary, NC, USA, 2009.

## 3. Results

### 3.1. Nutritional Composition

Table 2 shows the proximate characterization of tortillas made with different nixtamalization methods and RC. No significant differences (*p* > 0.05) were found between the RC and CTs, whereas TPTs and OHTs had slightly lower protein levels. Lysine and tryptophan, the two most limiting amino acids in corn, were found at 0.15–0.214 g per 100 g and 0.02–0.041 g per 100 g, respectively. The fat content was significantly higher in RC, followed by the TPTs and OHTs, whereas CTs exhibited the lowest fat value. The total dietary fiber content did not change with the OH method, whereas the traditional tortillas and CTs had a remarkable reduction in the total, insoluble, and soluble fiber. CTs and TPTs lost an average of 36.8% of total fiber and 23.3% of insoluble fiber, while OHTs did not lose insoluble fiber. CTs showed 90.7% less soluble fiber, whereas higher levels were found in TPTs (6.81%) and OHTs (120%). As expected, calcium was significantly increased (*p* < 0.05) in the TPTs and OHTs, showing values 4 times higher than RC and 2.65 higher than the CTs.

### 3.2. Protein Profile of Tortillas

**Figure 1:**
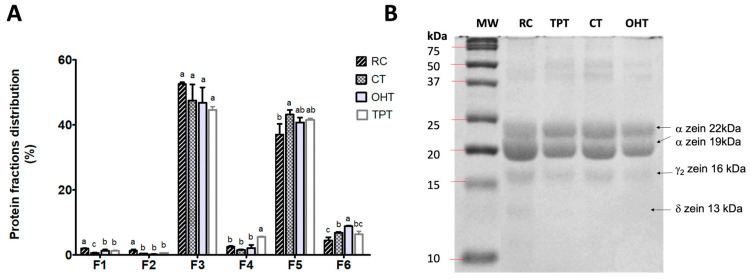
The protein profile and fraction distribution of raw corn and tortillas processed using traditional processing and ohmic heating. (**A**) The protein percentage of globulins and salt-soluble albumins (F1), albumins (F2), α-, β-, and δ-zeins (F3), γ-zeins and glutelin-like proteins (F4), glutelins (F5), and non-extractable (insoluble) protein residue (F6). RC: raw corn flour; CTs: commercial tortillas; TPTs: traditional-process tortillas; OHTs: tortillas processed using ohmic heating. (**B**) Electrophoretic profile of proteins (only F3 fraction is shown) showing the bands for α-, β-, and δ-zeins. Different letters indicate significant differences among groups for each protein fraction calculated using a Tukey test (*p* < 0.05).

The proportion of each protein fraction is shown in Figure 1A. Prolamins, including α-, β-, and δ-zeins (F3), as well as glutelins (F5), were the most abundant fractions, followed by the residue of insoluble proteins (F6). Globulins (F1) and albumins (F2) were the least abundant proteins in all samples. We observed a higher amount of residue of insoluble proteins (F6) in the tortillas processed using OH. For the remaining protein fractions, no significant changes were observed among tortillas produced using the different treatments. Since no differences were found in the SDS-PAGE, only the α-, β-, and δ-zeins (F3) analysis is shown. The electrophoresis pattern depicted in Figure 1B shows a similar band intensity for TPTs and OHTs, but they were less intense than the RC. The protein profiles showed bands around 19 and 22 kDa, which correspond to the α-zeins, 16 kDa corresponding to the β-zeins, and around 13 kDa possibly corresponding to the δ-zeins only in the RC sample [38].

### 3.3. Biological Assay

#### 3.3.1. Food Intake, Food Efficiency, and Body Weight Changes

**Figure 2:**
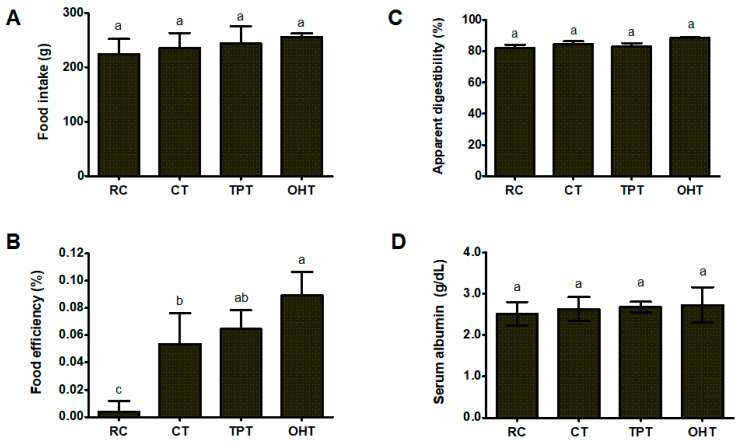
Changes in food intake (**A**), food efficiency (**B**), apparent digestibility (**C**), serum albumin (**D**) of rats fed tortilla-based diets made with different processes. RC: raw corn flour; CTs: commercial tortillas; TPTs: traditional-process tortillas; OHTs: tortillas processed using ohmic heating. Bars with different letters indicate significant differences between the experimental groups as determined using a Tukey test (*p* < 0.05).

The results obtained in this study for each experimental group are shown in Figure 2. The mean food intake (Figure 2A) was 239.86 g during the study, with no significant differences (*p* > 0.05) between the different groups. Although the rats consumed the same amount of food, the food efficiency (Figure 2B) showed important variations in the following order OHTs > TPTs > CTs > RC.

#### 3.3.2. Protein Performance

Protein digestibility (PD) varied from 82.07 to 88.52 g/100 g between the groups that were fed tortillas, while the casein group had a PD value of 95.65 g/100 g (Figure 2C). No significant differences were observed between the tortilla diets, although the OHT diet had a 3% higher digestibility than the other diets. Figure 2D shows that the serum albumin values in this study were not significantly different (*p* > 0.05) among the treatment groups. Except for the casein group, which showed normal values (3.6 ± 0.15 g/dL), all diets produced serum albumin values in the range of 2.63–2.73 g/dL, which is classified as moderated malnutrition [39].

**Figure 3:**
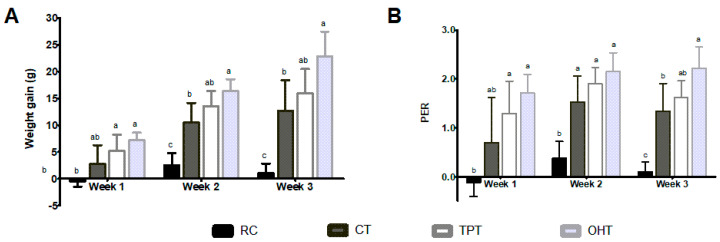
Changes in weight gain (**A**) and protein efficiency ratio (PER) (**B**) for rats fed tortilla-based diets made with different processes. RC: raw corn flour; CTs: commercial tortillas; TPTs: traditional-process tortillas; OHTs: tortillas processed using ohmic heating. Bars with different letters indicate significant differences between the experimental groups as determined using a Tukey test (*p* < 0.05).

The weekly growth performance of each experimental group is depicted in Figure 3A. Rats fed with OHTs and TPTs had the greatest weight gain, followed by CTs. After three weeks, the OHT group gained 22.82 ± 5.37 g in total. This value was not statistically different from the 15.94 ± 4.54 g gained by the TPT group but was higher than the 12.72 ± 5.63 g increase in the CT group. During the experiment, rats from the casein group had normal growth with an average weight gain of 101.3 g/week.

In contrast, the RC diet produced a significant weight loss in the first week (−0.44 ± 1.03 g) and a modest total weight gain (1.04 ± 1.82 g) by the end of the study. We observed a 1.8 times higher increase in the OHT group compared with the CT group, whereas this increase was 1.5 times higher than the TPT group. The PER of the experimental groups is shown in Figure 3B. Except for the CD (reference PER value = 2.5), the group fed the OHT diet had the highest protein performance (1.74), which remained constant during the study. For the remaining groups, the growth curve exhibited a maximum PER during the second week (TPT = 1.5, CT = 1.2, RC = 0.29); and thereafter, the growth rate decreased significantly to a final PER of 1.2, 1, and 0.07 for TPTs, CTs, and RC, respectively. The results obtained using the PD assay were positively correlated with the PER values (r = 0.68, *p* = 0.0015).

### 3.4. Bone Characteristics

**Figure 4:**
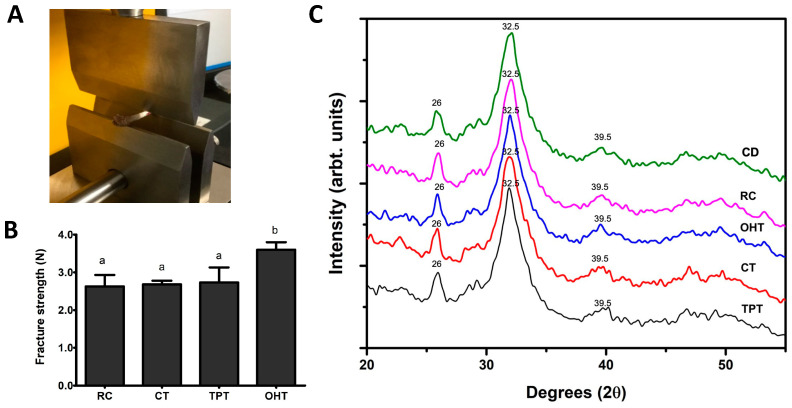
Fracture strength and X-ray diffraction pattern of the rat femur bones. The instrument configuration to perform the cutting test (**A**) and the fracture strength (**B**) and X-ray diffraction pattern (**C**) of the femurs from rats fed different diets. RC: raw corn flour; CTs: commercial tortillas; TPTs: traditional process tortillas; OHTs: tortillas processed using ohmic heating. Mean values with different letters in the same row were significantly different as determined using a Tukey test (*p* < 0.05).

Figure 4A shows the equipment configuration for the sample analysis. The results of the fracture test are depicted in Figure 4B for the different treatments. The fracture strength of the bones from rats fed OHTs was significantly higher than that of the other treatments, whereas RC, CTs, and TPTs had similar strength values. Figure 4C shows the X-ray diffraction pattern (XRD) of the femurs from the different study groups. Three main peaks were found at 2θ = 26, 2θ = 32.5, and 2θ = 39.5 that correspond to the 002, 211, and 130 planes, respectively. These results corroborated the presence of hydroxyapatite according to the International Center for Diffraction Data (ICDD card No. 01-084-1998). Except for the CD, which showed the highest intensity at the 32.5 peak, OHTs had the maximum intensity count (1099), whereas RC showed the lowest intensity count (924).

**Figure 5:**
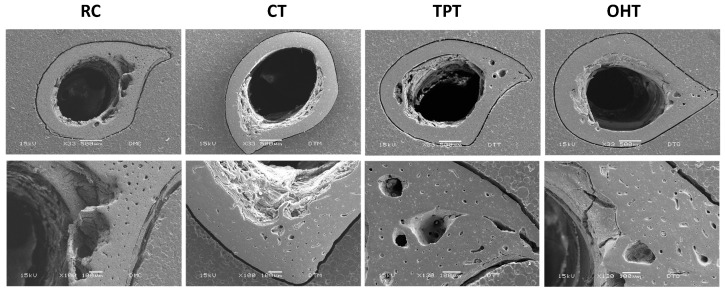
Scanning electron microscopy images showing transverse cuts of femurs from rats fed with different tortilla diets. RC: raw corn flour; CTs: commercial tortillas; TPTs: traditional process tortillas; OHTs: tortillas processed using ohmic heating.

The SEM images for transverse cuts of the femurs are shown in Figure 5. As expected, the group fed the CD showed dense, thick bones (images not shown). However, for comparison purposes, we show only the micrographs of the bones from the rats fed the tortilla and RC diets. Among the treatment groups, bones from the OHT group exhibited greater thickness and less porosity on the inner surface.

## 4. Discussion

The environmental impact of making tortillas has been well documented in the literature. Several alternative processes aim to reduce the negative effects of the nixtamalization method, often altering the sensory attributes or nutritional value of the tortillas [14,40] Given that tortillas are an important part of the diet in certain populations, new processes must provide at least the same nutritional benefits as traditionally prepared tortillas.

According to Castro-Muñoz et al. [11], the volume of wastewater (nejayote) generated in México reached 14.4 million L per year in 2015. Using OH technology to produce tortillas allowed us to reduce the excess water and eliminate the alkaline effluents generated using the traditional nixtamalization process. The process of producing nixtamalized corn flours using ohmic heating uses hydrated corn grains, as opposed to excessive water usage in traditional nixtamalization. This step prevents the generation of effluents and eliminates the washing step.

In our previous work, we reported the best conditions to obtain nixtamalized corn flours using OH, with adequate techno-functional properties to create tortillas [23,26]. The nutritional value of the tortillas processed with these flours showed a similar proximate composition, preserved their bioactive content, and had an increased dietary fiber content [23]. Furthermore, *“masa”* had a high water absorption capacity and an adequate tortilla yield, and the tortillas had an adequate inflation index and rollability [26].

The protein values are consistent with those reported in other publications for tortillas produced using different processing methods, with values ranging between 7.81 and 9.0 g/100 g [22,40,41]. The lysine and tryptophan contents were reduced after nixtamalization processing, as previously reported for tortillas investigated with different methods [22,42]. In our study, the lysine content was 0.18 g/100 g for TPTs and 0.15 g/100 g for OHTs, which indicates a 15–30% loss for this compound, while the tryptophan values were not significantly different (0.02 g/100 g). The lysine content in the CTs did not change compared to the RC, and a 17% reduction in tryptophan was observed; however, it is worth noting that commercial flours are commonly enriched with amino acids for commercialization. It is known that during nixtamalization and tortilla making, the lysine and tryptophan contents are affected by temperature, and about 18.60% lysine and 21.47% tryptophan are lost during the traditional process [43], whereas nearly 30% lysine and 70% tryptophan are lost during other alternative processes such as ultrasonic-bath and infrared heating [12].

Given the potential structural effects of OH on proteins, specific amino acid contents, such as lysine and tryptophan, might be influenced more significantly compared to TPTs, potentially impacting protein quality. In our investigation, we observed that although OH technology led to reduced levels of these amino acids in tortillas, the concentrations remained similar to those found in traditional tortillas. Moreover, we did not find an impact on protein availability and performance. In the biological assay, both the TPT and OHT groups exhibited superior weight gain and PER compared to the CT group (Figure 3). Given that industrial nixtamalization often removes these nutrients, the need for lysine and tryptophan enrichment in commercial tortillas becomes essential. However, comprehensive studies are necessary to confirm the depth of this enrichment’s impact on CT protein availability.

Regarding the fat values, some differences were found compared to other studies. Gaytán-Martínez et al. [22] reported higher fat values in tortillas prepared using OH (3.42–4.44 g/100 g), processed in a batch heater, and similar values obtained in TPTs (3.42 g/100 g). These differences may be attributed to the different methods used. With our OH method, the continuous feeding of corn flour appears to cause changes in the food structure that decreases the fat content, possibly due to saponification reactions after adding Ca(OH)_2_ [44]. Additionally, the formation of amylose–lipid complexes has been reported during the alkaline cooking of corn [45], which affects the extraction and quantification of lipids.

OHTs and RC had the highest total dietary fiber content, including insoluble and soluble fiber, indicating that the OH effectively preserves fiber during thermo-alkaline cooking, as previously observed by Ramirez-Jiménez et al. [23]. The most abundant fiber fraction was the insoluble fraction, which was significantly higher in the OH process. The soluble fiber was highest in the OHTs, followed by the TPTs, which may be due to the formation of resistant starch, particularly amylose–lipid complexes (known as type 5 resistant starch) during the nixtamalization process, a phenomenon that has been previously reported for corn [46,47]. These complexes appear when starch is heated and subsequently slowly cooled, a phenomenon observed in processes like nixtamalization, causing alterations in starch crystalline structure and digestibility [47].

Resistant starch is considered part of the non-digestible fraction of food, which, alongside fiber, confer benefits to gut health by enhancing local microbiota diversity and fostering the production of short-chain fatty acids. These compounds have been associated with satiety, hypoglycemic, and anti-obesity effects [48].

Since there is no washing after nixtamalization with the OH method, fiber is not lost during cooking and washing water, preserving its potential health benefits. Regarding the increase in calcium content in TPTs and OHTs, our findings are in agreement with other publications that report that the nixtamalization process allows for calcium diffusion inside the corn grain, increasing its content and bioavailability in tortillas.

The proportion and band pattern of the assessed protein fractions agrees with previous reports for raw and nixtamalized corn flours [49,50,51]. TPTs had the highest content of γ-zeins and glutelin-like proteins (F4), whereas this fraction was reduced in OHTs. The thermal and alkaline conditions used for nixtamalization decreased the abundance of zeins in the α-, β-, and δ-zeins (F3) fraction for TPTs and OHTs.

It is known that the nixtamalization process and further cooking of tortillas decrease the solubility of certain protein fractions, mainly albumins, prolamins, and globulins, which in turn, increase the residue of the insoluble proteins [50,51]. This might explain the higher amount of the residue of insoluble proteins (F6) in the OHTs, suggesting an effect of the temperature and electric field strength that deserves further investigation. This effect was confirmed in the protein pattern of the α-, β-, and δ-zeins (F3) shown in Figure 1B, where the less abundant proteins are present in the tortilla samples. Faint bands between 37 and 50 kDa suggest the presence of dimers [35] that are more intense in the tortilla samples, possibly due to the thermal and alkaline effects.

Earlier reports have found similar protein profiles, which correlate with changes in solubility. Glutelins exhibited increased solubility as an effect of alkaline cooking and steeping time. Enhanced protein solubility has been associated with higher accessibility to digestive enzymes, which in turn, might increase digestibility and nutritional value [3,4,50]. Moreover, glutelins have higher nutritional quality compared to the other fractions, thus contributing to the enhancement of tortilla nutritional value.

Food intake and food efficiency are two measurements that indicate the growth performance of weaning rats. In this study, the OHT and TPT diets exhibited the highest food efficiency, suggesting higher nutrient digestibility and bioavailability of tortillas processed using these methods. This may have benefits for populations that consume tortillas on a regular basis, improving adult nutrition and child growth. In other studies, a 1.7–2.0-fold higher efficiency was reported for rats supplemented with different protein sources compared with those with no protein supplementation [52]. Moreover, weight gain in the OHT and TPT groups was greater than the 19 g reported by Martínez-Flores et al. [4] but lower than the 41.14 g observed by Maya-Cortés et al. [5] in rats fed a corn diet, although the latter was a longer study. Apart from the carbohydrate influence on weight gain, protein intake has also been shown to affect weight gain due to the improved bioavailability of proteins. This was documented in a study with rats fed diets containing transgenic rice high in free lysine [53]. The PD values fall within the reported range for tortillas in the literature [2,54]. Although the OHT group showed a 3% higher digestibility than the other diets, we did not find significant differences between the groups. Other studies have shown that nixtamalization enhanced the amino acid profile of corn by increasing the solubility and digestibility of high-quality proteins such as glutelins [4]. This was confirmed in the tortillas processed using different alkaline-heating techniques. An average digestibility increase of 10% was observed after the corn was nixtamalized [12]. Moreover, tortillas prepared with the artisanal method exhibit higher digestibility values than those prepared with commercial corn flour [54]. Our study showed that the OH-assisted nixtamalization of tortillas over a short preparation time has the same effect on the nutritional values and PD as the traditional nixtamalization applied over a longer preparation time (~2 h).

PER has been considered an easy and reliable method to assess protein performance in animal models. This measurement indicates protein quality, which has been linked to growth performance. Our results showed no differences between the OH and traditional tortillas but an improvement in protein efficiency was noted when compared with CTs. The PER observed in our study for OHTs was lower than the PER (1.81) observed for tortillas obtained using ecological nixtamalization [5] and showed higher values than those reported for tortillas with an added soybean flour source (PER = 1.64) [55] and for tortillas obtained using extrusion (PER = 1.25) [4]. To confirm the nutritional status of the animals, we measured serum albumin, which is an important biomarker for protein metabolism in animal models. Rats fed with RC, CTs, and TPTs showed lower serum albumin values, whereas the OHT diet was close to the lower limit of mild malnutrition (2.73 g/dL). This reflects the inadequate protein nutritional status of rats fed with tortillas alone, which was expected since the protein supply was 7 g/100 g. Nevertheless, our results were higher than those reported by Maya-Cortés et al. [5] for rats fed tortillas for 28 days. The positive correlation between the PER and serum albumin suggests that the improved protein performance provided with OH and TP diets is strongly related to its digestibility. However, further studies on protein bioavailability are required to validate this finding. It has been reported that OH increases the digestibility of proteins due to alterations in the three-dimensional structure of proteins. The combined thermal and electrical effects of OH induce the denaturation of proteins through the unfolding and disruption of covalent bonds, thereby improving PD [56,57]. A recent study assessed the degree of hydrolysis of whey protein after applying OH (150 V/cm at 50 Hz for 5 and 15 s) in an in vitro digestibility model [58]. The higher degree of hydrolysis (and thus higher digestibility) was dependent on the processing time and was attributed to the disruption of the covalent bonds and more hydrophilic peptides. The slightly higher PD and PER of tortillas produced using OH might be attributed to these phenomena. Since we did not observe significant differences between the OH and TP diet in growth performance, digestibility, and PER, we confirmed that the nutritional value of tortillas produced using both methods is similar.

Tortillas are an important source of calcium for many Latin American populations, given the enhanced content and bioavailability of this mineral [7,9,10]. In this study, we analyzed rat femur bones as an indirect measure of calcium bioavailability. Bones are mainly composed of hydroxyapatite Ca_10_(PO4)_6_(OH)_2_, a form of calcium that confers crystallinity and strength to the bones and contributes to the structural integrity of bones and the process of bone healing. The presence and relative abundance of hydroxyapatite can be assessed using XRD. We observed peaks at 2θ = 26, 2θ = 32.5, and 2θ = 39.5, which are consistent with hydroxyapatite formation measured using XRD. The OHTs exhibited a similar hydroxyapatite diffraction pattern as TPTs. This suggests that the availability of calcium was similar for these diets since calcium was used for the formation of hydroxyapatite in the bones of the rats. This result is of importance, as it is one of the primary benefits of traditional nixtamalization. On the other hand, we also found that the bones from rats fed with the OHT diet had a higher fracture resistance. This potentially holds positive health implications, as heightened fracture resistance in rats consuming OHTs could contribute to improved long-term bone health and fracture prevention. The fracture strength of the bones from rats fed the TPT and CT diets was not statistically different from those of rats fed the RC diet. Since there was large variability within the samples, these results should be confirmed using a future study.

Finally, from the SEM images, we observed that the group fed the TPTs had thinner bones than the OHT group, with a larger porous area on the inner portion of each bone. The CT and RC groups had an important reduction in thickness and the presence of large cavities at the inner edges of the bones. This was expected for the RC group since the non-nixtamalized corn has less bioavailability, especially regarding calcium [59]. The results obtained in the cutting test agree with the data from the XRD and SEM analysis, indicating that the OHTs provided a slightly higher fracture resistance, crystallinity, and bone thickness. Other authors have observed similar results in rats fed diets with tortillas processed using TP and extrusion methods, concluding that crystallinity is proportional to bone density and higher fracture strength, which makes a bone more resistant [4]. Considering that tortillas constitute a primary calcium source in Mexico and certain Latin American nations, introducing a product that enhances bone strength and overall health could significantly enhance the nutritional well-being of these communities.

## 5. Conclusions

In our study, we demonstrated that tortillas created using OH technology had a similar protein profile and PER and a higher PD than that of the traditionally processed tortillas (TPTs). Moreover, calcium performance was improved in the OHT diet, with increased crystallinity, resistance to fracture, and bone thickness. The nutritional composition of OH tortillas showed similar values for protein, lysin, tryptophan, and fat compared to the traditional tortillas, and a higher content of dietary fiber, particularly insoluble fiber.

With these findings, we can state that OH is a suitable alternative technology for tortilla production. Apart from its technological and ecological advantages, it also provides a product with similar (and in some cases better) nutritional characteristics than traditional tortillas. Further studies are required to evaluate the bioavailability of amino acids in the OH tortillas and elucidate the increase in PD and PER observed in this study. Furthermore, the specific effect of OH on the protein structure and digestibility is also an issue that has not been studied in food. In summary, OH technology has the potential to reduce the gap between nutritional value and health implications, while considering the environmental impact. This is a big step toward making better and more sustainable food choices.

## Figures and Tables

**Table 1:** Nutritional composition of the experimental diets.

Component (g/100 g)	CD	RC	CT	TPT	OHT
Moisture	7.21 ± 0.07 ^b^	9.22 ± 0.07 ^a^	6.61 ± 0.07 ^c^	6.37 ± 0.03 ^d^	6.23 ± 0.06 ^d^
Ash	2.55 ± 0.06 ^a^	2.50 ± 0.01 ^a^	2.51 ± 0.07 ^a^	2.62 ± 0.03 ^a^	2.65 ± 0.04 ^a^
Fat	7.50 ± 0.06 ^a^	5.07 ± 0.14 ^b^	4.85 ± 0.23 ^b^	4.70 ± 0.03 ^b^	4.87 ± 0.20 ^b^
Protein	20.61 ± 0.30 ^a^	6.92 ± 0.05 ^d^	7.08 ± 0.07 ^c,d^	7.62 ± 0.01 ^b^	7.41 ± 0.07 ^b,c^
Total dietary fiber	5.69 ± 0.17 ^d^	8.16 ± 0.33 ^b^	6.95 ± 0.21 ^c^	6.72 ± 0.39 ^c^	10.83 ± 0.48 ^a^
Insoluble dietary fiber	5.03 ± 0.14 ^d^	7.82 ± 0.32 ^b^	6.46 ± 0.11 ^c^	6.21 ± 0.32 ^c^	10.03 ± 0.30 ^a^
Soluble dietary fiber	0.66 ± 0.03 ^a,b^	0.34 ± 0.00 ^b^	0.49 ± 0.10 ^b^	0.51 ± 0.07 ^a,b^	0.80 ± 0.18 ^a^
Carbohydrates	64.30 ± 0.28 ^d^	77.68 ± 0.13 ^b^	79.08 ± 0.29 ^a^	78.72 ± 0.39 ^a^	75.01 ± 29 ^c^

CD: casein diet, RC: raw corn flour diet; CT: commercial tortilla diet; TPT: traditional-process tortilla diet; OHT: a diet of tortillas processed using ohmic heating. Mean values with different letters in the same row were significantly different as determined using a Tukey test (*p* < 0.05).

**Table 2:** Nutritional composition of raw corn and tortillas produced using different methods.

Component (g/100 g)	RC	CTs	TPTs	OHTs
Moisture	9.29 ± 0.31 ^a^	6.66 ± 0.07 ^c^	4.21 ± 0.31 ^b^	6.25 ± 0.01 ^c^
Ash	1.20 ± 0.05 ^c^	1.32 ± 0.01 ^b^	1.50 ± 0.10 ^a^	1.48 ± 0.07 ^a^
Fat	4.43 ± 0.18 ^a^	2.45 ± 0.23 ^c^	3.09 ± 0.05 ^b^	2.73 ± 0.24 ^b,c^
Protein	8.68 ± 0.45 ^a^	8.30 ± 0.14 ^a^	7.62 ± 0.10 ^b^	7.46 ± 0.05 ^b,c^
Lysine	0.214 ± 0.01 ^a^	0.221 ± 0.01 ^a^	0.18 ± 0.01 ^b^	0.15 ± 0.01 ^c^
Tryptophan	0.041 ± 0.01 ^a^	0.034 ± 0.01 ^a^	0.02 ± 0.01 ^b^	0.02 ± 0.01 ^b^
Calcium	0.56 ± 2.00 ^c^	0.96 ± 2.00 ^b^	2.55 ± 2.00 ^a^	2.56 ± 1.00 ^a^
Total dietary fiber	10.04 ± 0.13 ^a^	7.65 ± 0.34 ^b^	7.53 ± 0.22 ^b^	10.72 ± 0.42 ^a^
Insoluble dietary fiber	9.49 ± 0.01 ^a^	7.60 ± 0.32 ^b^	6.95 ± 0.39 ^b^	9.52 ± 0.20 ^a^
Soluble dietary fiber	0.543 ± 0.12 ^b,c^	0.05 ± 0.07 ^c^	0.58 ± 0.28 ^b^	1.20 ± 0.22 ^a^
Carbohydrates	76.35 ± 0.39 ^c^	81.27 ± 0.42 ^b^	83.58 ± 0.10 ^a^	82.08 ± 0.12 ^b^

RC: raw corn flour, CTs: commercial tortillas, TPTs: traditional-process tortillas, OHTs: tortillas processed using ohmic heating. Mean values with different letters in the same row were significantly different as determined using a Tukey test (*p* < 0.05).

## Data Availability

The data used to support the findings of this study can be made available by the corresponding author upon request.

## Supplementary Materials

The following supporting information can be downloaded at: https://www.mdpi.com/article/10.3390/foods12183327/s1, Table S1. Formulation of the experimental diets containing RC and tortillas.
