# Supplementary material for: Sustainable Process for Tortilla Production Using Ohmic Heating with Minimal Impact on the Nutritional Value, Protein, and Calcium Performance
Source: Foods. 2023 Sep 5;12(18):3327. doi: 10.3390/foods12183327 (PMC10530181; doi:10.3390/foods12183327)
Supplement: Supplementary file 1 [file foods-12-03327-s001.zip › foods-2551234-supplementary.pdf]

Supplementary material

Sustainable process for tortillas production using ohmic heating with minimal impact on their nutritional value, protein, and calcium performance

Aurea K. Ramírez-Jiménez[1a], Rubén Cota-López[2a], Eduardo Morales-Sanchez[2], Marcela Gaytán-Martínez[3*], Héctor Eduardo Martínez-Flores[4], María de la Luz Reyes-Vega[5], Juan de Dios Figueroa-Cardenas [6]

Table S1. Formulation of the experimental diets containing corn flour and tortillas.

Ingredient (g/kg)	CD	RC	CT	TPT	OHT
Starch	535	45	-	19	-
Sucrose	100	100	94	100	-
Casein	200	-	-	-	-
Cellulose	50	-	-	-	-
Soybean oil	70	15	29	24	23
Minerals	35	25	24	23	21
Vitamins	10	10	10	10	10
RC	-	805	-	-	-
CT	-	-	843	-	-
TPT	-	-	-	824	
OHT	-	-	-	-	946

CD: Casein diet; RC: raw corn flour; CT: commercial tortillas; TPT: traditional process tortillas; OHT: tortillas processed by ohmic heating.
